# In-cell single-molecule FRET measurement of cytosolic RAF proteins to investigate the structural states and kinetics among them

**DOI:** 10.3389/fmolb.2025.1718018

**Published:** 2025-12-10

**Authors:** Kenji Okamoto, Yasushi Sako

**Affiliations:** Cellular Informatics Laboratory, Pioneering Research Institute, RIKEN, Wako, Japan

**Keywords:** single-molecule FRET, ALEX, RAF, protein conformation, live-cell measurement

## Abstract

The structure of a protein is closely linked to its function. Many proteins undergo conformational changes while working in living cells. Consequently, proteins in various structural states coexist in the native cellular environment. Understanding the structural heterogeneity of proteins in living cells is essential for understanding the kinetics of protein reactions. Single-molecule Förster resonance energy transfer (smFRET) is a powerful tool for probing the structure of biomolecules at the single-molecule level. Confocal smFRET measurements, which obtain the smFRET distribution of freely diffusing single molecules, have been successfully applied to cytosolic proteins in living cells. Previous studies on CRAF, a member of the RAF kinase family, revealed the coexistence of at least three conformational states and critical interactions with 14-3-3 proteins. In this study, we applied the method to, in addition to wild-type (WT) CRAF, to mutants at important sites, and to co-expression with other proteins related to RAF activation. The detailed analyses comparing those results suggest the presence of a fourth minor conformational state of CRAF in addition to the previously identified three major states. This fourth state may be related to RAF dimers. Supported by a newly introduced burst intensity analysis, we also found that the three major components can be classified into two groups: two interconvertible components and one independent component. Furthermore, in-cell smFRET measurement of wild-type BRAF, another RAF family member, revealed that its structural distribution consists primarily of a single species, which seemingly corresponds to the lowest FRET component among the three structural states of WT-CRAF. This finding suggests that BRAF has a fundamentally different structural and regulatory mechanism than CRAF.

## 1 Introduction

Understanding how proteins behave within the complex environment of living cells is a fundamental goal of molecular and cellular biophysics. In the cytoplasm, proteins continuously interact with a variety of molecular partners, many of which are unknown and cannot be systematically controlled or eliminated through experimental manipulation. Therefore, to gain a complete picture of protein structure, function, interactions, and kinetics in this native context, it is essential to observe proteins in their unperturbed, physiological state.

Fluorescence-based techniques are a powerful means of probing protein behavior in living cells. Advances in super-resolution microscopy have revealed subcellular structures with unprecedented detail (Radmacher et al., 2025). Fluorescence correlation spectroscopy (FCS) enables the extraction of diffusion coefficients, providing information about molecular size, interactions, and complex formation (Kim et al., 2007). On the other hand, Förster resonance energy transfer (FRET) provides direct access to intramolecular distance information and, consequently, to protein conformations. When performed at the single-molecule level, FRET can reveal heterogeneous structural distributions that are otherwise obscured in ensemble measurements (Lerner et al., 2021).

**FIGURE 1:**
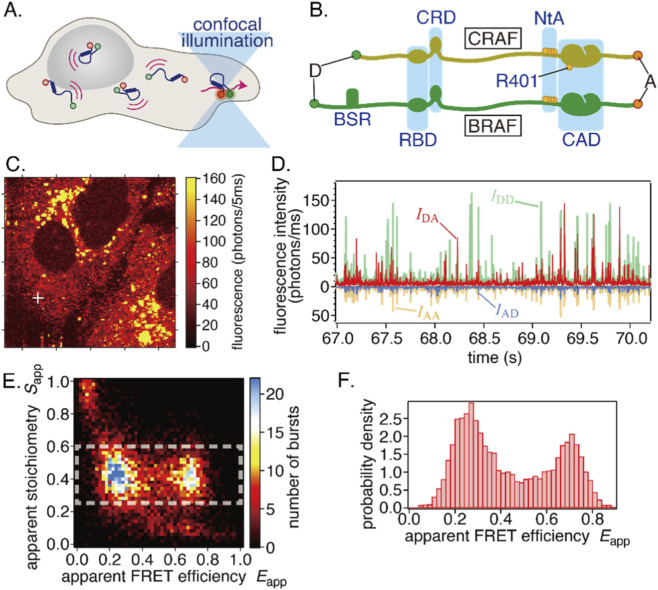
**(A)** Principle of in-cell smFRET measurements. Fluorescence is only detected when a molecule passes through the focus of the excitation light. **(B)** Structure of CRAF and BRAF proteins. Donor (D) and acceptor (A) dyes for FRET measurements are attached to the N- and C-termini, respectively. **(C–F)** Representative results from an in-cell smFRET measurement. **(C)** Confocal fluorescence image of HeLa cells. The white cross marks the focal position used for the subsequent measurements. The scan area is 50 × 50 µm^2^. **(D)** Four fluorescence time traces were acquired with two excitation colors and two detection channels, showing successive fluorescence bursts. **(E)** A two-dimensional histogram of stoichiometry *S*_app_ versus FRET efficiency *E*_app_ computed from single bursts. **(F)** An smFRET histogram for a single cell, constructed from the bursts within the white dashed rectangle in **(E)**.

Single-molecule fluorescence burst measurements, which detect fluorescence from individual molecules passing through the confocal volume transiently (Figure 1A), have been widely applied to *in vitro* studies. At sufficiently low concentrations, each fluorescence burst corresponds to a single molecule. This allows the FRET efficiency to be calculated on a per-burst basis, providing a single-molecule FRET (smFRET) distribution. Combining smFRET with the alternating laser excitation (ALEX) technique enables removal of photobleached or unlabeled species (Kapanidis et al., 2004). Recently, burst-based smFRET measurements have successfully been extended to living cells (König et al., 2015; Okamoto et al., 2020; Okamoto and Sako, 2023). Using genetically encoded fluorophores, GFP as a donor in combination with synthetic dyes bound to a HaloTag as an acceptor, has made it possible to measure FRET from proteins expressed *in situ*.

Rapidly accelerated fibrosarcoma (RAF) kinases are key components of the RAS-mitogen-activated protein kinase (MAPK) signaling pathway (Lavoie and Therrien, 2015). This family primarily consists of CRAF and BRAF, while ARAF plays only a minor role. The two major isoforms share similar structural and functional properties. While early structural models of the kinase domains were provided by X-ray crystallography (Hatzivassiliou et al., 2010; Qin et al., 2012), more recent cryo-electron microscopy (cryo-EM) studies have revealed various RAF complexes: including those with 14-3-3 of BRAF (Kondo et al., 2019; Park et al., 2019; Fiesco et al., 2022; Park et al., 2023; Lavoie et al., 2025) and CRAF (Dedden et al., 2024; Ryan et al., 2024; Jang et al., 2025), as well as CRAF associated with the HSP90-CDC37 chaperone complex (García-Alonso et al., 2022; Oberoi et al., 2022; Jaime-Garza et al., 2023), which had been suggested decades ago based on co-immunoprecipitation experiments (Wartmann and Davis, 1994). Further, smFRET studies have indicated that CRAF can adopt a mixture of two distinct closed conformations (Okamoto and Sako, 2023). Nevertheless, the precise architecture of these conformations and how they change during activation remain unresolved. While phosphorylation at the N-terminal acidic (NtA) motif and dimerization are thought to be involved, the sequence of structural events underlying RAF activation remains partially understood.

In this study, we use in-cell smFRET measurements to examine the structural distribution of cytosolic RAF proteins in their native cellular environment. This approach enables us to capture the conformational states and kinetics of RAFs among them under physiological conditions. In addition to wild-type (WT) CRAF, we obtained the smFRET distributions under conditions that can affect RAF structure, including mutants and co-expression of other proteins. Since the phosphorylation of the NtA motif is closely related to RAF activation, phospho- or dephospho-mimic mutants of the NtA motif may enhance or diminish conformational changes related to activation. A mutation at the dimer interface could alter the structural distribution if dimers are formed. Co-expression of AKT enhances phosphorylation at S259, which may increase the closed autoinhibitory conformation. Co-expression of RAS may enhance activation, including basal activation. As a result, we identified a fourth conformational state in addition to the previously reported three major components. Detailed fitting analyses, supported by the newly introduced burst intensity analysis, revealed that the three major components fall into two groups: two interconvertible components and one independent component. Furthermore, the in-cell smFRET measurements of WT-BRAF, another RAF family member, revealed a fundamentally different structural distribution than that of CRAF, suggesting distinct functions or regulatory mechanisms.

## 2 Results

### 2.1 The procedure and an example of in-cell smFRET measurement

Target RAF proteins were introduced into live HeLa cells via transient cDNA transfection, as described in the Materials and Methods section. The RAF proteins were labeled with GFP (FRET donor) at the N terminus and with tetramethylrhodamine (TMR) (FRET acceptor) at the C terminus via a HaloTag (Figure 1B). Due to variation in RAF expression among cells, we first identified a cell with an appropriate concentration of labeled RAF via camera imaging before measurement. Once identified, we acquired a confocal fluorescence image to define the cell’s position and shape (Figure 1C). Then, we positioned the focus at a point within the cell (marked by a cross in Figure 1C) and acquired four fluorescence time series with two excitation colors and two detection channels using ALEX (Figure 1D). The resulting signals comprised successive fluorescence bursts, each corresponding to a single molecule. For each burst, we calculated the apparent FRET efficiency *E*_app_ and apparent stoichiometry *S*_app_. A typical two-dimensional *E*_app_-*S*_app_ histogram is shown in Figure 1E. Since *S*_app_ reflects the donor/acceptor labeling stoichiometry of a single molecule, molecules that are doubly labeled with a donor and an acceptor are centered along the *S*_app_ axis. We then constructed the smFRET histogram from bursts within a specified *S*_app_ range (0.25 ≤ *S*_app_ ≤ 0.6; dashed rectangle in Figures 1E,F). This selection excludes singly labeled molecules, including bursts in which one of the dyes was photobleached or mislabeled. The example shown is WT-CRAF.

Previous studies (Okamoto et al., 2020; Okamoto and Sako, 2023) have identified at least three structural states in the typical smFRET distribution of CRAF, which consists of two peaks.

### 2.2 Average smFRET distributions

Because the number of bursts detected in a single living cell is limited, individual smFRET histograms often exhibit stochastic fluctuations. Therefore, we averaged smFRET histograms over multiple cells to enable comparison among measurement conditions.

**FIGURE 2:**
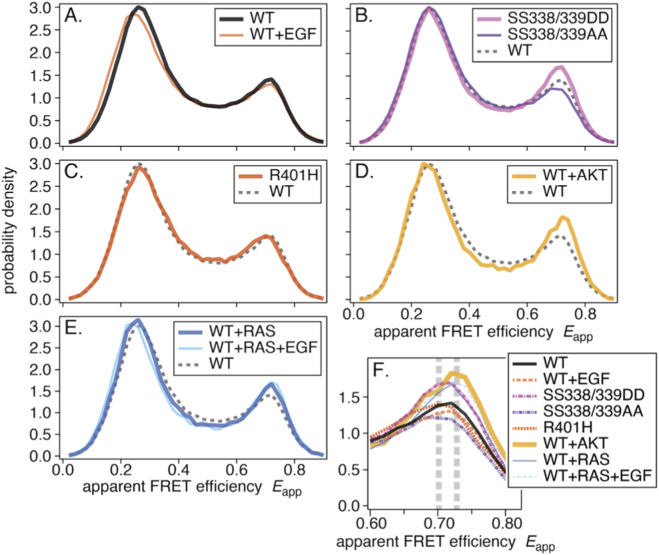
Average smFRET distributions: **(A)** WT and WT+EGF, **(B)** SS338/339DD and SS338/339AA, **(C)** R401H, **(D)** WT+AKT, and **(E)** WT+RAS and WT+RAS+EGF. In each panel, the dashed histogram shows WT for comparison. **(F)** The right peaks from the eight histograms. Two vertical dashed lines indicate two apparent peak positions.

Previous studies have reported averaged smFRET histograms of WT-CRAF before and after epidermal growth factor (EGF) stimulation (Okamoto et al., 2020; Okamoto and Sako, 2023). These studies showed a two-peak distribution with a minor EGF-induced change at the left peak (Figure 2A). Small intercellular variances suggested the regulation of structural distributions (Supplementary Figures S1A,B).

Mutations at certain essential sites, such as the cysteine-rich domain (CRD)/S259A/S621A, caused significant changes in the smFRET distributions (Okamoto and Sako, 2023). Since all of these sites are related to 14-3-3 binding, it was concluded that 14-3-3 binding is essential for CRAF.

Here, we present results for additional mutants and co-expression conditions.

#### 2.2.1 Phospho-/dephospho-mimic at NtA motif

CRAF has four consecutive phosphorylatable amino acids (SSYY, 338–341), in a linker region adjacent to the N-terminus of the kinase domain (Figure 1B). These amino acids are called the NtA motif, and their phosphorylation is thought to be involved in activation (Barnard et al., 1998; Mason et al., 1999; Chaudhary et al., 2000). In BRAF, the corresponding residues (446–449) are SSDD. There are two negatively charged residues and serines are constitutively phosphorylated (Mason et al., 1999), contributing to BRAF’s high basal activity (Marais et al., 1997). We generated phospho-mimic (SS338/339DD) and dephospho-mimic (SS338/339AA) mutants at the CRAF NtA motif.

The averaged smFRET histograms (Figure 2B) closely resemble WT-CRAF, with a discernible difference in the right peak. Individual histograms with variances are shown in Supplementary Figures S1C,D.

#### 2.2.2 Mutation at the dimer interface

RAF can form homo- and heterodimers among isoforms. The arginine 401 residue of CRAF is located at the dimer interface, and the R401H substitution has been shown to disrupt dimerization (Rajakulendran et al., 2009; Freeman et al., 2013). We generated the CRAF R401H mutant and measured its smFRET in cells.

The averaged smFRET histogram (Figure 2C) was nearly identical to that of WT-CRAF. This suggests that most CRAF may not form dimers in quiescent cells, though it is still possible that FRET does not change between monomers and dimers. The histogram with variance is shown in Supplementary Figure S1E.

#### 2.2.3 Co-expression of inhibitory kinase AKT

AKT, also known as protein kinase B (PKB), phosphorylates serine 259 of CRAF (Zimmermann and Moelling, 1999), which promotes 14-3-3 binding and forms an autoinhibitory conformation. We prepared membrane-localized and constitutively activated AKT (Kohn et al., 1996) and coexpressed it with WT-CRAF.

The averaged smFRET histogram shows slight deviations over a broad range of *E*_app_ relative to WT-CRAF (Figure 2D). Supplementary Figure S1F shows the histogram with variance.

#### 2.2.4 Co-expression of upstream signaling protein RAS

RAS is a membrane-anchored protein located directly upstream of RAF in the RAS-MAPK signaling pathway. In overexpression experiments, RAS is often co-expressed with RAF to prevent limiting upstream signaling. We measured the smFRET of WT-CRAF with RAS co-expression before and after EGF stimulation.

The averaged smFRET histograms were similar to those of WT-CRAF, with slight deviations at both peaks and in the inter-peak region (Figure 2E). Supplementary Figures S1G,H show the individual histograms with variances.

#### 2.2.5 Comparison of the right peaks

As described above, we compiled averaged smFRET histograms for eight conditions: mutations and co-expression with or without EGF stimulation. Unlike mutations at the 14-3-3 binding sites, drastic changes in the shape of the histograms were not observed. Slight shifts of the left peak are consistent with EGF-induced population shifts. Other than this, discernible differences were seen in the shapes of the right peaks.

Figure 2F plots the right peaks for all eight conditions. Significances of differences in smFRET distributions between experimental conditions were tested and shown in the Supplementary Table . Different peak positions are apparent, as indicated by the dashed vertical lines. These differences suggest that the right peak comprises at least two components.

### 2.3 Global fitting analysis

**FIGURE 3:**
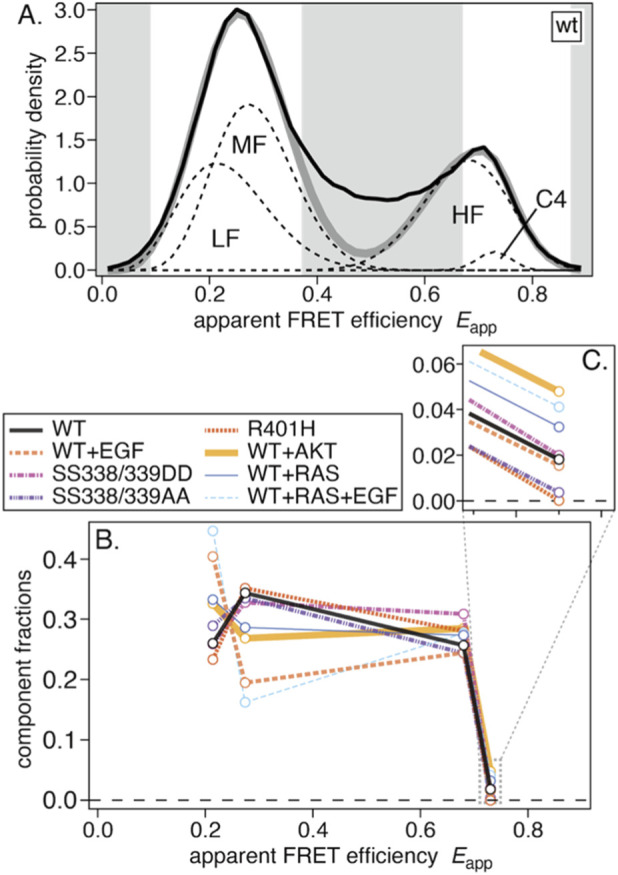
Results of the global-fitting analysis. **(A)** Average smFRET distribution for WT with fitted components LF, MF, HF, and C4. Gray bands denote the fitting mask used to exclude data points from the analysis. **(B)** The resulting fractions of the four components for the eight measurement conditions. **(C)** An enlarged view of the C4 fractions.

Previously, we conducted global fitting analyses to compare populations of structural components across conditions (Okamoto and Sako, 2023). Assuming three components—two contributing to the left peak and one to the right—we obtained satisfactory results. However, the present data suggest that the right peak may consist of two components. Therefore, we repeated the global fitting analysis on eight averaged histograms, assuming four components with two in each of the left and right peaks (Figure 3A). Each component was represented by a single beta distribution, and a fitting mask was introduced to fit only the peak positions.

All averaged smFRET histograms were successfully fitted by four components. From low to high FRET, the low-FRET (LF), middle-FRET (MF), and high-FRET (HF) components closely resembled those in our previous work (Okamoto and Sako, 2023). An additional minor fourth component (C4) was also identified. The fit results are summarized in Table 1, and component fractions are plotted in Figure 3B. Fitting residuals and condition-wise results are shown in Supplementary Figure S2.

**TABLE 1:** Results of the global-fitting analysis. *E*_*j*_ and *I*_*j*_ are globally fitted parameters representing the peak position and width, respectively.

		LF	MF	HF	C4
Index	*j*	1	2	3	4
Peak parameters	*E*_*j*_	0.21	0.27	0.68	0.73
	*I*_*j*_	24.8	35.1	32.7	201.0
Peak fractions	WT	0.249 ± 0.156	0.355 ± 0.156	0.254 ± 0.017	0.017 ± 0.008
	WT+EGF	0.397 ± 0.215	0.203 ± 0.210	0.242 ± 0.017	0.014 ± 0.008
	SS338/339DD	0.248 ± 0.154	0.339 ± 0.154	0.305 ± 0.018	0.018 ± 0.009
	SS338/339AA	0.279 ± 0.169	0.346 ± 0.169	0.241 ± 0.016	0.003 ± 0.008
	R401H	0.222 ± 0.143	0.363 ± 0.145	0.275 ± 0.016	0.000 ± 0.008
	WT+AKT	0.317 ± 0.182	0.278 ± 0.180	0.283 ± 0.023	0.046 ± 0.012
	WT+RAS	0.323 ± 0.186	0.297 ± 0.184	0.271 ± 0.019	0.031 ± 0.010
	WT+RAS+EGF	0.440 ± 0.234	0.170 ± 0.227	0.275 ± 0.021	0.039 ± 0.011

The sum of the component fractions for a given condition (mutant, co-expression, and ±EGF) does not equal one because some data points were excluded from the analysis. See Section 5.4 of the Materials and Methods for details. Errors are the estimated standard deviations of the fitting coefficients.

Compared with the previous analysis, the LF fraction was larger, likely reflecting differences in the datasets used for global fitting. EGF-induced population shifts from MF to LF (WT → WT+EGF and WT+RAS → WT+RAS+EGF) were also larger, whereas shifts from HF to LF were not observed as before.

The fractions of the C4 component (enlarged in Figure 3C) were small in all eight conditions but varied among them. Fractions were nearly zero for the R401H and SS338/339AA mutants but were higher upon co-expression of AKT or RAS.

### 2.4 Fitting components to individual cells

We used averaged smFRET histograms to estimate peak parameters because single-cell histograms contained too much stochastic fluctuation for reliable multi-parameter fitting. After determining the four peak positions and widths from the averages, we fitted only the component fractions for individual cells, keeping the positions and widths fixed.

**FIGURE 4:**
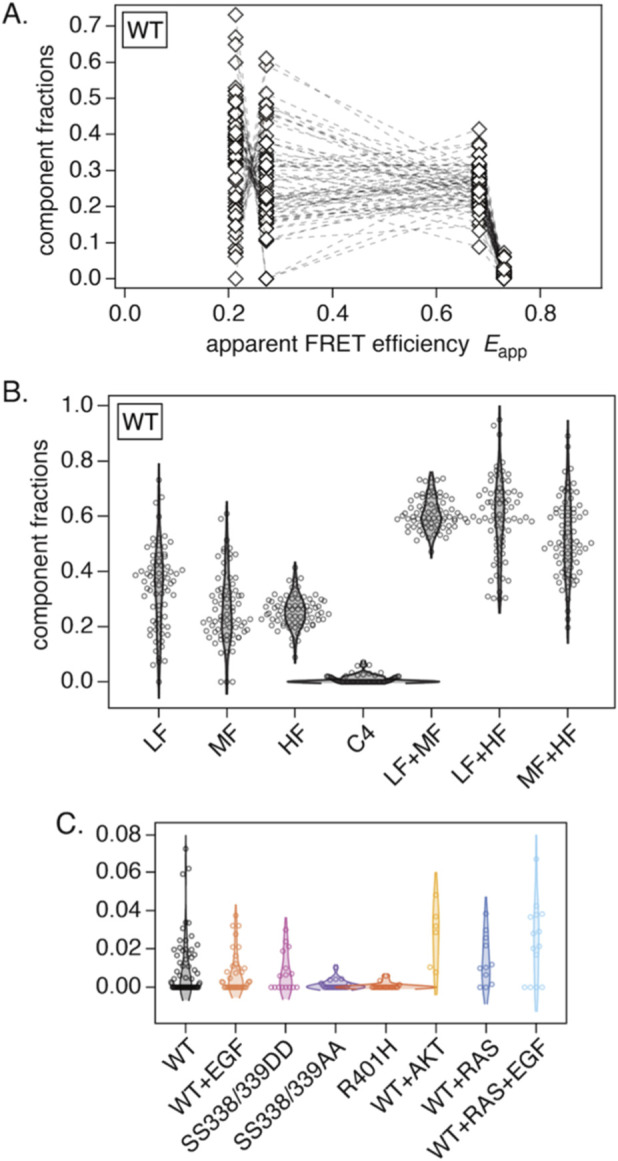
Results of fitting four peaks to individual cells. **(A)** Fitted fractions for WT, where the four component fractions for the same cell are connected. **(B)** Distributions of fractions for each component and for selected sums (LF + MF, LF + HF, and MF + HF) in WT. **(C)** Fitted fractions of the fourth component C4 across the eight measurement conditions.

The results for WT-CRAF are shown in Figure 4A, and the results for the other conditions are shown in Supplementary Figure S3. There was substantial variance for the LF, MF, and HF components, whereas the variance of the averaged histogram was relatively small (see Supplementary Figure S1A). Interestingly, anticorrelations were apparent between the LF and MF components, and, to a lesser extent, between the MF and HF components. To visualize this, we plotted the distributions of the fitted fractions in Figure 4B, including the four components and their summed combinations (LF + MF, LF + HF, and MF + HF). Compared with the LF and MF components, the HF component exhibited smaller variance. While the variances of LF and MF were relatively large when considered individually, their sum displayed smaller variance. A similar tendency was observed under other conditions (see Supplementary Figure S4). In all conditions, the HF components and the LF + MF sums have fractions of ∼0.3 and ∼0.6, respectively, suggesting regulation that keeps them comparatively constant.

#### 2.4.1 Fourth component

The smFRET distribution of a single species is represented by a beta distribution function with the parameters FRET efficiency *E*_*j*_ and mean burst intensity *I*_*j*_ (see Equation 1). Since a higher *I*_*j*_ represents a narrower peak, *I*_*j*_ can be considered a parameter that represents peak width. A peak with a lower *I*_*j*_ than the average of the experimentally detected burst intensities indicates a broader peak than the theoretical prediction, suggesting the structural fluctuations or multiple structures with slightly different FRET efficiencies within it (Okamoto and Sako, 2023). *I*_1_–*I*_3_ in Table 1 correspond to this case. Conversely, the C4 component with a high *I*_4_ value may represent a relatively fixed structure that is not necessarily similar to the HF component.

The distributions of C4 fractions obtained from single-cell fits are plotted in Figure 4C. Compared to WT-CRAF, the C4 fraction increased with co-expression of AKT or RAS and decreased significantly with the SS338/339AA and R401H mutations. Neither the SS338/339DD mutation nor EGF stimulation appeared to appreciably affect the C4 fraction. The identity of the C4 component is discussed in the Discussion section.

### 2.5 Burst intensity analysis

In ALEX measurements, two parameters are obtained from each burst: FRET efficiency *E* and stoichiometry *S*. Here, we also analyzed a third parameter, the burst intensity *I*.

*I* is defined as the number of photons within a single fluorescence burst. The value of *I* for an individual burst is stochastic because a fluorescent molecule undergoes a random walk through the excitation focus in addition to stochastic fluorescence emission. The residence time in the focus is random, and the excitation efficiency varies as the molecule diffuses due to the spatial profile of the excitation light. Therefore, direct comparison of *I* between individual bursts is not meaningful. However, *I* can be evaluated statistically and compared across species. For example, the mean *I* for a population of dimers should be approximately twice that of monomers.

**FIGURE 5:**
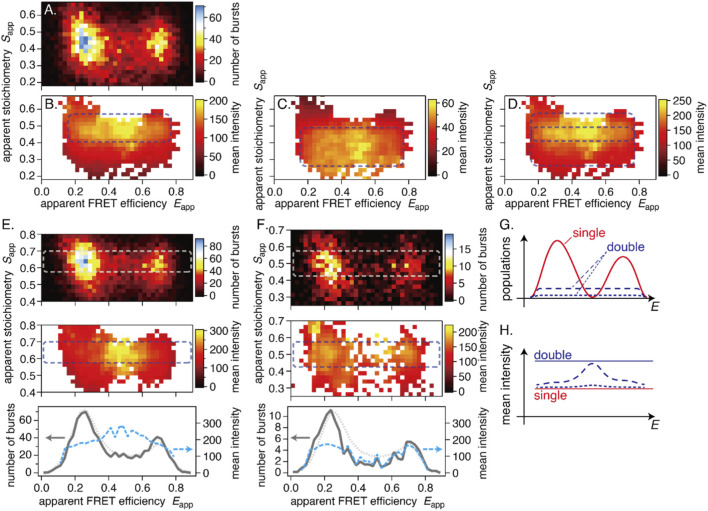
Example results of the burst-intensity analysis. **(A)** Burst distribution on the *E*-*S* map, **(B)** mean burst intensity of donor-excited fluorescence, **(C)** mean burst intensity of acceptor-excited fluorescence, and **(D)** their sum. **(E,F)** Examples of characteristic mean-intensity distributions. Top: burst distribution; middle: distribution of the summed mean burst intensity; bottom: cross-sections through the dashed rectangles. Gray solid lines are from top views, and blue dashed lines are from middle views. Dotted curves are the average smFRET distribution (scaled) of WT for comparison. **(E)** A cell with a high-intensity distribution in the inter-peak region. **(F)** A cell without a high-intensity distribution in the inter-peak region. **(G,H)** Conceptual illustration of burst composition in the smFRET histogram. **(G)** Burst distributions: most of the two-peak distribution consists of single bursts (red solid lines); double-burst events are fewer but broadly distributed (dashed and dotted lines). **(H)** The mean-intensity distribution: high-intensity features arise where the single-burst distribution is minimal and contributions from double bursts are substantial. The dashed and dotted lines represent the resulting mean intensity distribution for the case with few double bursts and almost no double bursts, respectively. Horizontal lines indicate the mean intensity of single and double bursts, respectively. All results are WT.

We constructed burst-intensity maps (Figures 5A–D), in which the average intensity within each bin was calculated as a weighted average of all bursts (see the Materials and Methods section and Supplementary Figure S5A). Examples include donor-excited intensities *I*_Dexc_ (Figure 5B), acceptor-excited intensities *I*_Aexc_ (Figure 5C), and total intensities (sum of them) (Figure 5D). The difference in absolute intensity between *I*_Dexc_ and *I*_Aexc_ arises from the difference in excitation strength because the donor and acceptor are excited at a ratio of approximately 3:1 to 4:1 within each ALEX cycle.

*I*_Dexc_ and *I*_Aexc_ are not uniformly distributed along the *S* direction. Figure 5B shows a high-intensity region at higher *S*_app_ (∼0.5) and a low-intensity region at lower *S*_app_ (<0.4). In contrast, Figure 5C shows a high-intensity region at lower *S*_app_ (∼0.3–0.4) and a low-intensity region at higher *S*_app_ (>0.5). Since variation in *S*_app_ reflects the numbers of donor and acceptor dyes, these patterns may indicate dimer formation. However, a similar tendency was observed for a double-stranded DNA (dsDNA) reference sample that does not dimerize (see Supplementary Figures S5E–I). We ultimately attributed the *S*-dependency of the mean burst intensities to an instrumental artifact (see Supplementary Text for details). One should be careful not to assume that variation in the mean burst intensity or a broadened distribution along the *S*-axis directly indicates dimer formation.

#### 2.5.1 High-intensity burst distribution between peaks

We examined the burst intensity distributions of all cells. There were variations from cell to cell, but, unfortunately, we could not find characteristic patterns that imply dimer formation and are specific to the measurement conditions.

Regardless of the conditions, however, we found a frequently observed feature: a high-intensity burst distribution is formed in the inter-peak region (Figure 5E). A similar feature is also visible in Figures 5B–D. This feature was not universal. For instance, in a cell with a very low concentration of labeled CRAF, no high-intensity distribution was evident between peaks (Figure 5F). We discuss the potential meaning of this high-intensity distribution in the Discussion section.

### 2.6 BRAF

**FIGURE 6:**
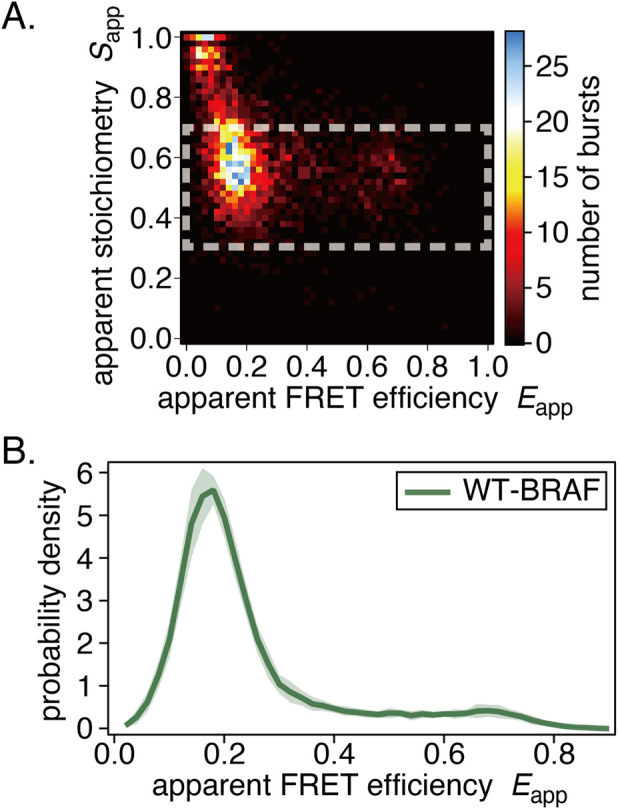
Results of in-cell smFRET measurements of BRAF. **(A)** Example *E*-*S* two-dimensional histogram. **(B)** The average smFRET histogram, shown with the standard deviation, exhibits a single-peak distribution.

We performed ALEX-smFRET measurements of WT-BRAF. Example results are shown in Figure 6. The smFRET distributions exhibited a single peak (Figures 6A,B), which is similar to the S621A or CRD mutants of CRAF. This suggests that WT-BRAF natively adopts a half-open or full-open conformation (Okamoto et al., 2020; Okamoto and Sako, 2023).

Further mutation or co-expression studies on BRAF were not performed because changes in smFRET distributions were not expected.

## 3 Discussion

Using the ALEX technique, we measured the smFRET distributions of CRAF proteins in live cells under various conditions, including the mutations SS338/339DD and SS338/339AA, the R401H mutation, and the co-expression of AKT or RAS. Detailed analyses of smFRET histograms and fluorescence-burst statistics—including newly introduced individual-cell fitting or burst intensity analyses—provided new insights into CRAF kinetics in living cells.

### 3.1 The average smFRET distributions

The average smFRET histograms exhibit similar overall shapes across all eight conditions. However, slight differences are evident in the position of the left peak, as well as in the height and shape of the right peak. The fact that these similarities are robust under various perturbations, including mutation, co-expression of other proteins, and EGF stimulation, implies that CRAF state populations are tightly regulated.

### 3.2 Global fitting analyses

We performed a global fit of the average smFRET histograms and successfully fit them with four components. A clear outcome was the shift of the population from the MF to the LF components upon EGF stimulation. Similar behavior has been reported previously, though the shifted fraction was smaller (Okamoto and Sako, 2023). We also found no evidence of a shift from the HF to the LF components. Additionally, we identified a fourth component.

To further characterize the four populations, we fit individual-cell histograms with the four peak positions fixed. In our previous study, the MF and HF components appeared equivalent despite their distinct FRET efficiencies because they responded similarly to mutations at the 14-3-3 binding sites (Okamoto and Sako, 2023). However, noticeable differences emerged in the present study. The variances of the populations were relatively small for the HF component and the sum of the LF and MF components, whereas the variances of the LF and MF components were large when considered separately. These results suggest that intracellular CRAF can be classified into two fundamentally different species. The LF + MF (LMF) component may constitute one species that interconverts between two structural states depending on cellular conditions or EGF stimulation. This species should be related to signal transduction because it responds to EGF. The MF component may be stored for signal transduction and convert to the LF component upon activation. The LF component represents the activated form and is observed even in quiescent cells via basal activation. Note that the LF component can include open conformations that do not interconvert to the MF state. In contrast to MF and HF, which form discernible FRET peaks and thus imply more compact, recurring structures, LF probably aggregates bursts with undetectable FRET, encompassing a range of more open conformations. Conversely, the HF component may represent an independent species. It may be stored for other, currently unspecified functions. Notably, CRAF has functions beyond signal transduction, such as autophagy (Toifl et al., 2025), regulation of mitosis and tumor progression (Mielgo et al., 2011), and DNA repair (Advani et al., 2015). The LMF and HF species may correspond to different complex compositions because CRAF interacts with 14-3-3 proteins (Tzivion et al., 1998) and forms complexes with HSP90 and CDC37 (Wartmann and Davis, 1994; García-Alonso et al., 2022).

This study first identified the fourth component, C4. Although C4 lies at the right peak, it does not necessarily share the same conformation or function as HF. It may be related to RAF dimerization, as the R401H or SS338/339AA mutation nearly eliminates C4. R401 resides at the dimer interface and the R401H mutation disrupts dimerization (Rajakulendran et al., 2009; Freeman et al., 2013). In the crystal structure of CRAF dimers, the negatively charged residues of the NtA motif and the arginines on the αC-helix, including R401, are closely positioned (Hatzivassiliou et al., 2010). Their interaction has been reported to stabilize dimers (Hu et al., 2013; Jambrina et al., 2016; Takahashi et al., 2017). Small fractions of C4 under the other six conditions may represent basally formed dimers. Co-expression of AKT or RAS may enhance basal dimer formation. Although the phosphorylation of the NtA motif is believed to be associated with RAF activation, SS338/339DD does not increase the C4 fraction, suggesting that aspartic acid substitutions are insufficient as phosphomimics. Additionally, if C4 corresponds to activated dimers, it is puzzling that EGF stimulation does not increase its fraction and that the LF component also appears to include activated dimers. One possible explanation is that activated dimers adopt a distinct conformation from basally formed dimers.

### 3.3 Burst intensity analyses

To further examine the ALEX results, we analyzed the burst intensity *I*, which provides additional information from each fluorescence burst, alongside the FRET efficiency *E* and the stoichiometry *S*. A statistical evaluation of *I* should be able to detect molecular interactions that alter intensity, such as dimer formation.

A typical pattern observed in the burst-intensity distributions was the presence of lower- and higher-intensity regions along the *S*-axis in both the donor- and acceptor-excited intensity maps, albeit with opposite trends (Figures 5B–D). Since a similar pattern was observed with a dsDNA sample (see Supplementary Figures S5F–I), we attributed it to an instrumental artifact (see Supplementary Material).

Additionally, we observed a characteristic pattern in which high-intensity bursts populate the region between the two major peaks (Figure 5E). Bursts in this region are usually considered state transitions within a single burst (Gopich and Szabo, 2007). However, it is difficult to explain why only high-intensity bursts would undergo state transitions. There are two possible interpretations.

First, two bursts consecutively pass through the laser focus and are detected as a single burst (see Supplementary Figure S6B). Such double-bursts have a mean intensity approximately twice that of single bursts. Their distribution is nearly flat between the two peaks in the *E* histogram (dashed line in Figure 5G), reflecting the various contributions of LMF and HF, including double-bursts composed of LMF-LMF or HF-HF bursts in the peak regions. The number of double-bursts should be much smaller than that of single bursts (red solid line in Figure 5G). When computing the mean intensity at each *E* value, the influence of double bursts is limited in the peak regions by averaging with the abundant single bursts. By contrast, in the inter-peak region, where single-burst counts are minimal, the mean intensity is disproportionately influenced by double-bursts. Consequently, the mean-intensity profile exhibits a high-intensity peak between the peaks (dashed line in Figure 5H). The experimental data follow this pattern (bottom panel in Figure 5E). However, such a high-intensity peak in the inter-peak region was not always observed. Figure 5F shows an example. In this cell, the concentration of labeled CRAF was low and the number of detected bursts was smaller than in other examples (Figure 5A or Figure 5E). Accordingly, the probability of double-burst detection is markedly reduced (dotted line in Figure 5G). Example time traces for the cells in Figures 5E,F are shown in Supplementary Figures S6C,D, respectively. The burst distribution is smaller than average (solid and dotted lines in Figure 5F), resulting in a nearly flat mean-intensity profile (dotted line in Figure 5H).

Another interpretation is that only dimers transition between the LMF and HF states. This interpretation is consistent with concentration dependence (Figures 5E,F), since more dimers are formed at higher concentrations. However, this hypothesis contains an inconsistency. If dimers increase the mean intensity in the inter-peak region, then the base signal of monomers must be low, nearly zero. However, since the concentration of unlabeled endogenous CRAFs may be more than comparable with that of labeled CRAFs (Materials and Methods 5.3.4), a significant proportion of the detected dimers should be formed with an unlabeled CRAF and be detected as monomers. This would result in an unignorable base signal between the peaks. Additionally, such dimers should be distributed in the inter-peak region of population histograms (gray solid lines in Figure 5F), which would prevent populations from dropping below the average level. Therefore, for this hypothesis to hold, one of the following two assumptions must be true. First, a labeled CRAF forms a dimer selectively with another labeled CRAF. Second, only the intermolecular FRET changes during the transition, while the intramolecular FRET stays constant, so that the FRET of half-labeled dimers does not change. Neither of these assumptions appears plausible. Therefore, we support the first double-burst hypothesis.

The double-burst hypothesis consistently explains the presence of a high mean intensity and, at the same time, suggests that the single-burst population is small in the inter-peak region. This also means that there is little spontaneous interconversion between the LMF and HF states. Previously, we inferred spontaneous state transitions between the left and right peaks using dynamic analyses, such as FRET-two-channel kernel-based density distribution estimator (2CDE) (Okamoto et al., 2020) and burst variance analyses (BVA) (Okamoto and Sako, 2023). However, these observations could also be explained by double-burst detection. In principle, photon signals are indistinguishable between structural transitions in a single burst and double-burst detections unless burst intensity is evaluated (see Supplementary Figures S6A,B). Therefore, double-burst detection should be minimized because it can mislead inferences about RAF kinetics. However, achieving sufficiently low concentrations to avoid this issue is more challenging in in-cell experiments. See Section 5.3.4 of the Materials and Methods section for details.

In sum, our analysis of burst intensity did not reveal evidence of dimer formation. We did not detect a signature of the C4 component in the mean-intensity maps, although it may contribute to high-intensity features if it forms dimers. The LF component, which should include activated CRAFs, seems like monomers, too. However, these results do not rule out RAF dimer formation. Homodimers involving unlabeled endogenous CRAF and heterodimers involving A/BRAF are apparently detected as monomers, even if formed.

### 3.4 BRAF

For the first time, we present the smFRET distribution of WT-BRAF in living cells. Its distribution differs entirely from that of WT-CRAF and instead resembles that of open CRAF mutants (S621A, CC165/168SS), including the presence of a minor distribution at the HF position of CRAF (Figure 6) (Okamoto and Sako, 2023). While CRAF and BRAF are often assumed to behave similarly in terms of complex formation, activation, and functional output, our results suggest that their behavior in live cells can differ significantly.

### 3.5 Concluding model

**FIGURE 7:**
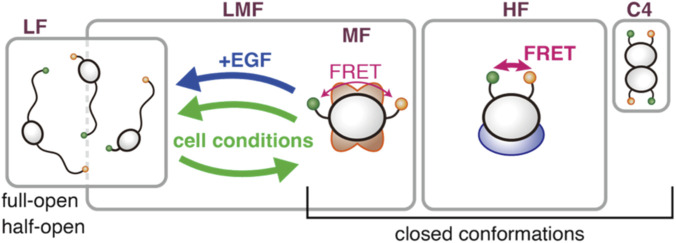
Concluding model. CRAF in cells comprises four components. The three major components, LF, MF, and HF, are partitioned into two species: an interconvertible LMF and an independent HF. The populations of the LMF and HF species are regulated to maintain a constant ratio between them. Depending on cellular conditions, the LF and MF components interconvert within the LMF species. EGF stimulation, in particular, shifts some of the MF toward the LF. Spontaneous transitions between the LMF and HF species do not occur. These two species may form distinct complexes with different partner molecules. The fourth, minor component, C4, may be related to RAF dimers.

In conclusion, we propose a renewed model of the intracellular structural states of CRAF (Figure 7). There are three major components (LF, MF, and HF) and one minor component (C4), which may be related to dimers. The three major components can be grouped into two species: interconvertible LMF and independent HF. LF may include other open conformations. The population fractions of the two species are regulated to remain constant at ∼0.6 and ∼0.3, respectively. The LMF species distributes between the LF and MF components depending on cell conditions or EGF stimulation, suggesting a relationship to signal transduction. The MF may serve as a pool that can be converted to LF upon signaling activation. Even under conditions such as nutrient starvation, where the cell may wish to conserve resources, a minimal level of signaling is still physiologically required; maintaining an MF pool could help secure this. Conversely, the HF species appears independent of the LMF and, consequently, of signal transduction. HF may be related to other, as yet unspecified, functions, such as autophagy, and may serve as a reservoir for them. There is little to no spontaneous transition between the LMF and HF species within the millisecond time scale detectable by burst measurement. This allows us to hypothesize that they form complexes with different compositions. However, redistribution can occur on a longer timescale, as suggested by population regulation. In that case, our previous conclusion that both the MF and HF components are bound to 14-3-3 (Okamoto and Sako, 2023) would need to be reconsidered. If one is bound to 14-3-3 and lost due to mutations, the other may be lost through the regulation that maintains the LMF:HF ratio, independently of 14-3-3. The similarity of BRAF to the LF, the activated state of CRAF, as well as its higher basal activity, suggests it is more specialized for kinase activity. It is possible that EGF-responsive CRAF regulates BRAF activity via heterodimerization or related mechanisms.

## 4 Conclusion

We have described in-cell smFRET measurements. Using a confocal microscope with the focus set on a point inside a living cell, we detect two-color fluorescence emitted by freely diffusing molecules within the cytoplasm. We calculate FRET efficiencies exclusively for doubly labeled single molecules using ALEX. The resulting smFRET histograms reveal heterogeneous structural distributions. We applied this technique to RAF proteins. We observed CRAF’s response to EGF stimulation, which indicates that RAF protein functionality is preserved and captured. The influence of overexpression, which often perturbs protein behavior, is minimized by the inherent requirement of fluorescence-burst measurements to use very low sample concentrations.

In this study, in addition to global fitting analysis on the average smFRET histograms, we introduced individual cell fitting and burst intensity analyses. The results of these detailed analyses are summarized in Figure 7. Additionally, we demonstrated that the structural distribution of BRAF is markedly different from that of CRAF, suggesting distinct behaviors, functionalities, and working mechanisms.

Single-molecule measurements in individual living cells allowed us to study the intercellular heterogeneity of the intracellular heterogeneous distributions of protein structures, which led to the elucidation of intracellular structural states and their interconversion kinetics. Although we could not detect RAF dimer formation by burst-intensity analysis in the present system, mapping mean intensity variation demonstrated sensitivity to fluorescence intensity changes. This indicates the ability to detect dimers if they are formed.

## 5 Materials and methods

### 5.1 Cell culture and expression in live cells

The protocol has been previously described (Okamot et al., 2020; Okamoto and Sako, 2023). In brief, HeLa cells were cultured in Dulbecco’s Modified Eagle Medium (high glucose) supplemented with L-glutamine (FUJIFILM Wako Chemicals Co., Ltd., Japan) and 10% fetal bovine serum. For all experiments, cells that transiently expressed the proteins of interest were used. Subconfluent cells (∼10% confluence) were transfected with the EGFP-RAF-HaloTag expression vector using Lipofectamine 3,000 (Thermo Fisher Scientific Inc.). To suppress the expression levels of the labeled proteins, the concentrations of the cDNA and P3000 reagents were reduced to 10%–20% of those in the standard protocol and the incubation time was shortened from 2–4 days to 3 h cDNAs for AKT and RAS were added according to the standard protocol.

The cells were starved overnight in Eagle’s minimum essential medium (EMEM; Nissui Pharmaceutical Co., Ltd., Japan) supplemented with 1% bovine serum albumin (BSA). Before measurement, the HaloTag was labeled with a ligand covalently attached to the fluorescent dye TMR (Promega Corp.). During measurements at room temperature, the cells were kept in EMEM supplemented with 1% BSA and 15 mM HEPES (pH 7.4) (Nacalai Tesque, Inc., Japan). For the +EGF condition, EGF was added to a final concentration of 100 ng/mL.

### 5.2 Plasmid construction

The protocols for preparing the cDNA encoding EGFP-CRAF-HaloTag have been previously described (Okamoto et al., 2020). Briefly, point mutations were introduced into the cDNA by site-directed mutagenesis using the QuikChange Lightning Site-Directed Mutagenesis Kit (Agilent Technologies, Inc.) and PrimeSTAR Max DNA Polymerase (Takara Bio Inc., Japan). All mutations were confirmed by DNA sequencing. Seven plasmids were constructed for this study. The WT-CRAF plasmid was the same as that used in previous studies (Okamoto et al., 2020; Okamoto and Sako, 2023).

The other CRAF mutants were derived from WT-CRAF. Two mutations were introduced at the NtA motif, the phosphorylation of which is considered essential for CRAF activation (Barnard et al., 1998; Mason et al., 1999; Chaudhary et al., 2000). In the phospho-mimic mutant SS338/339DD, the two serines were substituted with aspartic acids, and in the dephospho-mimic SS338/339AA, they were substituted with alanines. R401 is thought to be located at the interface of RAF homo- and heterodimers. The R401H mutant has been shown to disrupt dimer formation in vitro kinase-domain molecule experiments (Rajakulendran et al., 2009; Freeman et al., 2013). WT-BRAF were kindly provided by Drs. Robert Hayward and Richard Marais (Signal Transduction Team at the Cancer Research United Kingdom Centre for Cell and Molecular Biology at the Institute of Cancer Research in London, United Kingdom). For FRET measurements, EGFP and HaloTag were encoded at the N- and C-termini, respectively, in all RAF constructs.

For co-expression experiments, plasmids for additional proteins were prepared. AKT is a serine kinase for residue 259 in CRAF (Zimmermann and Moelling, 1999). The pleckstrin homology (PH) domain 4–129 of AKT was replaced with the SKSKPKDPSQRSD *src* myristoylation signal sequence, which localizes AKT to the plasma membrane and constitutively activates it (Kohn et al., 1996). pCMV-Ras (HRAS) was purchased from Clontech (TaKaRa, Ohtsu, Japan).

### 5.3 ALEX-smFRET measurement

#### 5.3.1 Apparatus

The apparatus has been described elsewhere (Okamoto et al., 2020; Okamoto and Sako, 2023). In brief, it is a confocal microscope assembled around a commercial microscope (Ti-E; Nikon Instech Co., Ltd., Tokyo, Japan). Two lasers, one with a wavelength of 488 nm (Sapphire 488–10 CDRH, Coherent, Inc., CA, United States) and the other with a wavelength of 561 nm (OBIS 561–100 LS, Coherent, Inc.), were used to excite the donor and acceptor dyes, respectively. Laser modulation for ALEX measurements was achieved using an acousto-optic modulator (TEAF3-0.45-0.7-S, Brimrose Corp., MD, United States) for the 488 nm light and digital modulation of the OBIS laser driver for the 561 nm light. A two-channel function generator (AFG3022B, Tektronix, Inc., OR, United States) generated the modulation signal. The beams were combined with a dichroic filter (LM01-503-25; IDEX Health and Science, LLC, NY, United States) and introduced into the microscope via a single-mode optical fiber. In the microscope, a dual-band dichroic filter (ZT488/561rpc, Chroma Technology Corp., VT, United States) directed the excitation light to the sample through an objective lens (CFI Apo TIRF 60XC Oil; Nikon). A dual-band emission filter (ZET488/561m, Chroma Technology Corp.) blocked residual excitation light. After donor and acceptor fluorescence were separated by another dichroic filter (540DCLP, Chroma Technology Corp.), avalanche photodiode (APD) photon-counting modules (SPCM-AQR-14/16, Excelitas Technologies Corp., MA, United States) detected the single photons. A bandpass filter (FF01-593/40–25, IDEX Health and Science, LLC) was placed before the APD for the acceptor detection channel. The two APD signals and the SYNC signal from the function generator were counted by a PCI-interface counter board (LPC-632104, Interface Corp., Hiroshima, Japan) on a PC. The sample was placed on a piezo scanner with closed-loop control (P-542.2CL, Physik Instrumente, Karlsruhe, Germany). A scientific complementary metal-oxide-semiconductor (sCMOS) camera (ORCA-Flash4.0V2+C11578-22C, Hamamatsu Photonics K.K., Shizuoka, Japan, or 01-PRIME-R-M-16-C-CP, Photometrics) was used to identify cells prior to ALEX measurements.

#### 5.3.2 Finding a cell to measure

In this study, fluorescently labeled sample proteins were introduced into living cells via genetic engineering. However, controlling the expression level of the sample proteins is difficult. Therefore, we had to identify a cell with an appropriate sample concentration for measurement among cells with various expression levels. We used a highly inclined and laminated optical sheet (HILO) illumination technique (Tokunaga et al., 2008) with 488 nm light and a highly sensitive sCMOS camera to find suitable cells with appropriately low concentrations that appeared faintly fluorescent.

#### 5.3.3 ALEX measurement procedure

Once an appropriate cell was identified using camera imaging, a fluorescence confocal image was acquired via stage scanning (Figure 1C). Then, the excitation light was focused on a point within the cell near the glass surface. This allows for the separate detection of fluorescence bursts while minimizing background.

For ALEX measurements, the excitation lights were alternated in 100 µs cycles. Fluorescence signals were recorded as photon arrival times with a time resolution of 100 ns. In typical ALEX measurements, the donor and acceptor excitation windows are of equal length. However, we used unequal excitation windows (typically 3:1 or 4:1) because donor-excited photons provide more information for quantifying FRET efficiency. In contrast, acceptor-excited photons only report on the fluorescent activity of the acceptor dye. The detected photons were classified into four types based on the excitation color (D or A) and the detection channel (D or A). The four fluorescence time series *I*_XY_, where X and Y denote excitation and detection, respectively, were reconstructed (Figure 1D). Background intensities were calculated for each of the four channels. We extracted fluorescence bursts from time-stamped photon streams using the all-photon-burst-search (APBS) method (Nir et al., 2006) with minor modifications.

Fluorescence photons detected from a diffusing molecule in the focused spot form a fluorescence burst (Figure 1D). Typically, 2,000–10,000 fluorescence bursts are collected during a 2–5 min measurement of an individual cell (actual numbers of detected and analyzed bursts are summarized in the Supplementary Data). At a sufficiently low concentration of the sample (typically 0.1–1 nM), single bursts correspond to single sample molecules. Then, the FRET efficiency *E*_FRET_ and stoichiometry *S* of single molecules can be calculated. *E*_FRET_ depends on the inter-dye distance *r* as

$$E_{\text{FRET}}\left(r\right)=\frac{1}{1+{\left(r/R_{0}\right)}^{6}}$$

with *R*_0_ being the Förster distance (i.e., the distance at which *E*_FRET_ = 0.5). *S* corresponds to the labeling ratio of the two dyes on a single molecule. Strictly, several compensation parameters are required to calculate the exact *E*_FRET_ and *S* from fluorescence signals (e.g., leakage between detection channels and differences in quantum yields or detection efficiencies). Here, we analyze the apparent FRET efficiency *E*_app_ and apparent stoichiometry *S*_app_ without those compensations:

$$E_{\text{app}}=\frac{I_{\text{DA}}}{I_{\text{DA}}+I_{\text{DD}}}$$

$$S_{\text{app}}=\frac{I_{\text{Dexc}}/f_{\text{Dexc}}}{I_{\text{Dexc}}/f_{\text{Dexc}}+I_{\text{Aexc}}/f_{\text{Aexc}}}$$

where *I*_Dexc_ = *I*_DA_ + *I*_DD_ and *I*_Aexc_ = *I*_AA_ + *I*_AD_. With the ALEX cycle width *τ*_cyc_ = 100 µs and donor- (acceptor-) excitation window widths *τ*_Dexc_ (*τ*_Aexc_), the donor (acceptor) excitation fractions are defined as *f*_Dexc_ = *τ*_Dexc_/*τ*_cyc_ (*f*_Aexc_ = *τ*_Aexc_/*τ*_cyc_).

#### 5.3.4 Concentration of target molecules

Sample concentration is a critical parameter in fluorescence-burst measurements. In vitro experiments, it is typically prepared at concentrations of ∼nM or sub-nM. If the concentration is too high, individual bursts cannot be resolved. As discussed in the Discussion, multiple bursts may be consecutively detected as a single burst. Therefore, it is safest to prepare samples at sufficiently low concentrations for *in vitro* experiments.

However, achieving very low concentrations in cells is challenging. First, expression levels are difficult to control by transient transfection (or even in stable cell lines) at very low concentrations. Second, it is difficult to find cells with extremely low concentrations, even when using a highly sensitive camera capable of single-molecule imaging, because diffusion of cytosolic molecules blurs fluorescence images. Additionally, a sufficient number of bursts for statistical analysis may not be detected in a single cell. Longer accumulation may be required, but this risks damaging the cells. Additionally, measurable molecules may be lost by photobleaching during measurement. Consequently, cells with higher concentrations than those used *in vitro* are often measured with a focus on the peripheral, thin region to decrease the apparent concentration. Consequently, double bursts occur more frequently than *in vitro* experiments.

CRAF is abundantly expressed, and its concentration is estimated to be ∼10 nM (Fujioka et al., 2006; Surve et al., 2019). It is difficult to estimate the actual concentration of introduced labeled RAFs in measured cells, but it may be in tens of nM range, even when higher than *in vitro* experiments. Thus, the influence of overexpression should be less significant. This is one advantage of in-cell ALEX-smFRET measurements.

### 5.4 Global fitting analysis

In previous work (Okamoto and Sako, 2023), the average smFRET histograms were successfully fitted with three components. Each component was represented by a beta distribution (Dahan et al., 1999):

$$f_{j}\left(x;I_{j},E_{j}\right)=\frac{x^{I_{j}E_{j}}\times {\left(1-x\right)}^{I_{j}\left(1-E_{j}\right)}}{B\left(I_{j}E_{j}+1,I_{j}\left(1-E_{j}\right)+1\right)}$$ (1)

where *j* indexes components, and *E*_*j*_ and *I*_*j*_ denote the FRET efficiency and the mean burst intensity of the *j*-th component, respectively; $B\left(a,b\right)=\int_{0}^{1}t^{a-1}{\left(1-t\right)}^{b-1}dt$ is the beta function. A fitting mask was introduced to exclude some data points because the inter-peak region typically represents bursts with state transitions within a burst and cannot be described analytically. Only data points in peak regions (*E*_app_ of 0.08–0.38 and 0.66–0.88) were used for fitting.

In this study, we conducted a global fitting analysis with four components. The masked smFRET distributions were fitted with

$$F_{i}\left(x;A_{i},I,E\right)=\sum_{j=1}^{4}A_{ij}f_{j}\left(x;I_{j},E_{j}\right)$$

where *i* indexes measurement conditions, **A**_*i*_ = {A_*i*1_, A_*i*2_, A_*i*3_, A_*i*4_} represents the component fractions, and **I** = {*I*_1_, *I*_2_, *I*_3_, *I*_4_} and **E** = {*E*_1_, *E*_2_, *E*_3_, *E*_4_} are the component widths and positions, respectively. *I*_*j*_ and *E*_*j*_ were global fitting parameters for the *j*-th component. **A**_*i*_ was fitted for each of eight conditions. The summation $\sum_{j=1}^{4}A_{ij}$ was not equal to unity because some data points were excluded by masking. First, the four components were globally fitted with six conditions without EGF stimulation. Next, the peak parameters for HF and C4 (positions *E*_3_ and *E*_4_, widths *I*_3_ and *I*_4_) were fixed, and the model was globally fitted to all eight conditions.

### 5.5 Burst intensity analysis

To investigate the influence of burst intensity, we aimed to visualize the distribution of burst intensities on a two-dimensional *E*-*S* coordinate system. First, we calculated the mean intensity of donor- and acceptor-excited bursts in each bin of the two-dimensional map. The results are shown in Supplementary Figures S5B–S5D. While the overall tendencies of the distributions are visible, the maps are jagged due to the insufficient number of data points to produce smooth distributions, a common occurrence in single-molecule experiments.

Next, we calculated the weighted mean burst intensity. The mean intensity in the *m*-th bin is calculated from all bursts, including those outside the target bin, with the weight *w*_*nm*_:

$${\langle I\rangle }_{m}=\frac{\sum_{n}^{N}w_{nm}I_{n}}{\sum_{n}^{N}w_{nm}}$$

where *n* indexes bursts, *N* is the total number of bursts, *I*_*n*_ is the intensity of the *n*-th burst, and $\sum_{m}^{M}w_{nm}=1$ , *M* is the total number of bins. Because the single-molecule fluorescence signal is stochastic, the detected *E* and *S* may deviate from the true values. This broadens the measured distributions according to the beta distribution. We defined the weight as the probability that the *n*-th burst at (*e*_*n*_, *s*_*n*_) is truly included in the *m*-th bin (positioned at (*e*_*m*_, *s*_*m*_), the center of the bin) (see Supplementary Figure S5A). According to Bayes’ theorem, *w*_*nm*_ can be calculated as

$$w_{nm}=\frac{p\left(n|m\right)p\left(m\right)}{\sum_{m}^{M}p\left(n|m\right)p\left(m\right)}$$

where *p* (*n*|*m*) is the posterior probability that the burst at (*e*_*m*_, *s*_*m*_) is detected at (*e*_*n*_, *s*_*n*_), which can be written using the distribution function in Equation 1.

$$p\left(n|m\right)=f_{j}\left(e_{n};I_{n}^{\text{Dexc}},e_{m}\right)\times f_{j}\left(s_{n};I_{n}^{\text{tot}},s_{m}\right)$$

where *I*_*n*_^Dexc^ and *I*_*n*_^tot^ are the donor-excited and total intensities of the *n*-th burst, respectively. The prior probability is defined based on the experimental data as

$$p\left(m\right)=\frac{n\left(m\right)}{N}$$

with *n*(*m*) being the number of bursts in the *m*-th bin.

Figures 5B–D show the weighted mean intensities calculated for the same cell as in Supplementary Figures S5B–S5D. Smoothed distributions are revealed while preserving the overall tendencies.

## Data Availability

The original contributions presented in the study are included in the article/Supplementary Material, further inquiries can be directed to the corresponding authors.
