# Supplementary material for: In-cell single-molecule FRET measurement of cytosolic RAF proteins to investigate the structural states and kinetics among them
Source: Front Mol Biosci. 2025 Dec 10;12:1718018. doi: 10.3389/fmolb.2025.1718018 (PMC12727557; doi:10.3389/fmolb.2025.1718018)
Supplement: Supplementary file 1 [file Supplementaryfile1.pdf]

Supplementary Material

1 Supplementary Text

1.1 *S*-dependent Distributions in Mean Intensity Distributions

1.1.1 Principle

We calculated the mean burst intensity distributions on the E_{app}-S_{app} map of the ALEX results and found that the mean intensity of the D:A=1:1 labeled molecules typically varies along the *S*-axis, as shown in Figure 5 of the main text. When an *S*-dependent distribution is generated, one might assume that ratios of donor (D) and acceptor (A) excitation efficiency vary among molecules. This occurs when there is a displacement in focal positions between the two excitation wavelengths (Supplementary Fig.S7A).

In our system, the excitation lights are coupled into the microscope through the same single-mode optical fiber. Residual chromatic aberration in the objective/relay optics could move the focal position and generate this displacement. Additionally, since the point-spread function (PSF) scales with wavelength, the D- and A-excitation PSFs differ in size. Even when their centroids coincide, the two PSFs do not perfectly overlap.

1.1.2 Experimental Validation by 3D Fluorescent Bead Imaging

We directly measured the focus offset by three-dimensional imaging of fluorescent beads (TetraSpeck^{TM} Microspheres; Thermo Fisher Scientific Inc.). *x*-*y* scans were performed with the sample stage, and *z* scans were performed using the microscope's Perfect Focus System. We alternated between two excitation colors at each voxel and acquired two 3D stacks.

The results are shown in Supplementary Figures S7C–H. The PSFs were imaged larger than the theoretical prediction because the bead's non-zero size was convoluted. The two foci were displaced by tens of nanometers in both the axial and transaxial directions. The difference in focus sizes could also be seen. The apparent tilt in the vertical sections (Supplementary Figs.S7F–H) is due to thermal drift during the relatively slow *z*-scanning process. The offset between PSFs changed with the objective lens, implicating objective chromatic aberration of the objective lens as a contributing factor.

1.1.3 Simulation

We tested the impact of chromatic focus differences with a simple simulation. We neglected diffusion and donor-acceptor spatial separation: we placed a two-dye emitter at a random point within a focus. Initially, the D- and A-excitation intensities were sampled from Gaussian PSFs. Then, we calculated the four fluorescence intensities (two excitations × two detections) considering FRET, spectral bleed-through, and direct excitation of A by D-excitation light. We drew photon counts from Poisson statistics and converted them to the apparent observables E_{app} and S_{app}, which were then plotted as two-dimensional histograms (Supplementary Figs.S8A, E, and I).

We assumed a 488-nm excitation PSF with σ_{xy} = 90 nm and σ_z = 170 nm, and scaled the 561 nm-PSF size by 561/488 ≒ 1.15. We imposed a 40 nm transaxial focus offset between the two excitations (Supplementary Fig.S7A). We assumed three FRET states (*E* = 0.25, 0.58, 0.85), a fluorescence bleed-through from the D- to the A-detection channel of 0.07, and a direct excitation efficiency of A by D-excitation of 0.10 to resemble the dsDNA results (Supplementary Figs.5G–I). Under these conditions, the simulated mean intensity map reproduced the qualitatively identical broadening and spatial distributions along the *S*-axis observed experimentally (Supplementary Figs.S8A–D).

Removing the transaxial offset while retaining the wavelength-dependent PSF sizes yielded narrower distributions. The pattern changed noticeably under A-excitation; however, an *S*-dependent distribution persisted (Supplementary Figs.S8E–H). When the PSFs were made perfectly identical (no focus offset or wavelength scaling), a similar, albeit weaker, trend remained, which was attributable to photon-counting stochastic noise (Supplementary Figs.S8I–L).

1.1.4 Summary

A spatial mismatch between the D- and A-excitation foci generates an apparent *S*-dependent distribution of mean burst intensity. In our current optical layout, from the single-mode optical fiber output to the sample, there is no mechanism to nullify this offset by independently aligning the beam paths, so the effect is unavoidable. The *E* coordinate should essentially remain unaffected.

Mitigating this effect would require independently adjusting the focus for each excitation color. To further match the PSF sizes, the effective NA after the objective (i.e., the pre-objective beam diameter) would also need to be tunable. With these two degrees of control, we expect the residual broadening to approach the photon-statistics limit shown in Supplementary Figures S8I–L.

2 Supplementary Table and Figures

2.1 Supplementary Table

	WT	WT+EGF	SS338/339DD	SS338/339AA	R401H	WT+AKT	WT+RAS	WT+RAS+EGF
WT			**	*		**	*	**
WT+EGF			***	*		***	**	***
SS338/339DD				***	**	*		
SS338/339AA						***	***	***
R401H						**	*	**
WT+AKT								
WT+RAS								
WT+RAS+EGF								

Supplementary Table. Significances of differences between two histogram groups were tested using the distance-based PERMANOVA (permutational multivariate analysis of variance) method. Two groups of histograms of individual cells at the right peak ($0.66 \leq E_{app} \leq 0.88$) were compared using the adonis2 function in the vegan package of R (ver. 4.5.2) with 100,000 permutations.

2.2 Supplementary Figures

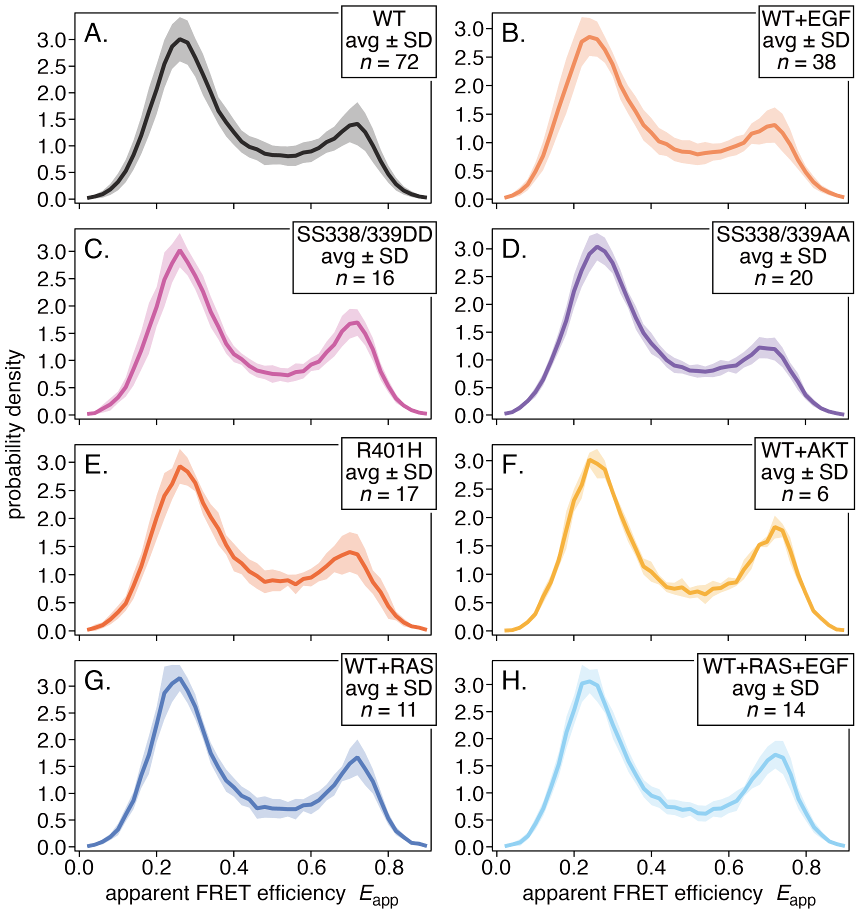

Supplementary Figure S1. The average smFRET histograms for **(A)** WT, **(B)** WT+EGF, **(C)** SS338/339DD, **(D)** SS338/339AA, **(E)** R401H, **(F)** WT+AKT, **(G)** WT+RAS, and **(H)** WT+RAS+EGF. *n* is the number of cells measured. Bands represent the standard deviations.

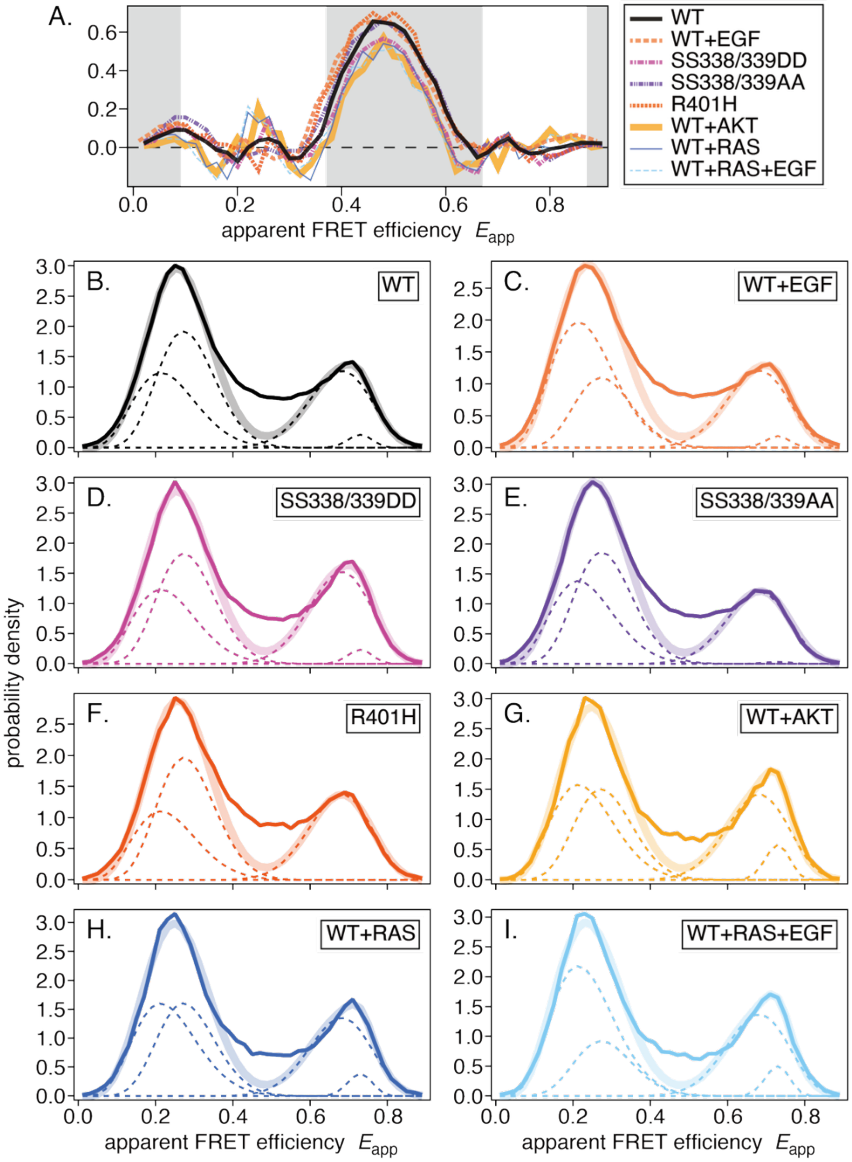

Supplementary Figure S2. Global fitting results. **(A)** Fitting residuals. Fitted curves for **(B)** WT, **(C)** WT+EGF, **(D)** SS338/339DD, **(E)** SS338/339AA, **(F)** R401H, **(G)** WT+AKT, **(H)** WT+RAS, and **(I)** WT+RAS+EGF. Solid lines are experimental results. Dashed lines represent fitted components, LF, MF, HF, and C4 from left to right. Thick light-colored solid lines are their sum. **(B)** is identical to Fig.3A of the main text.

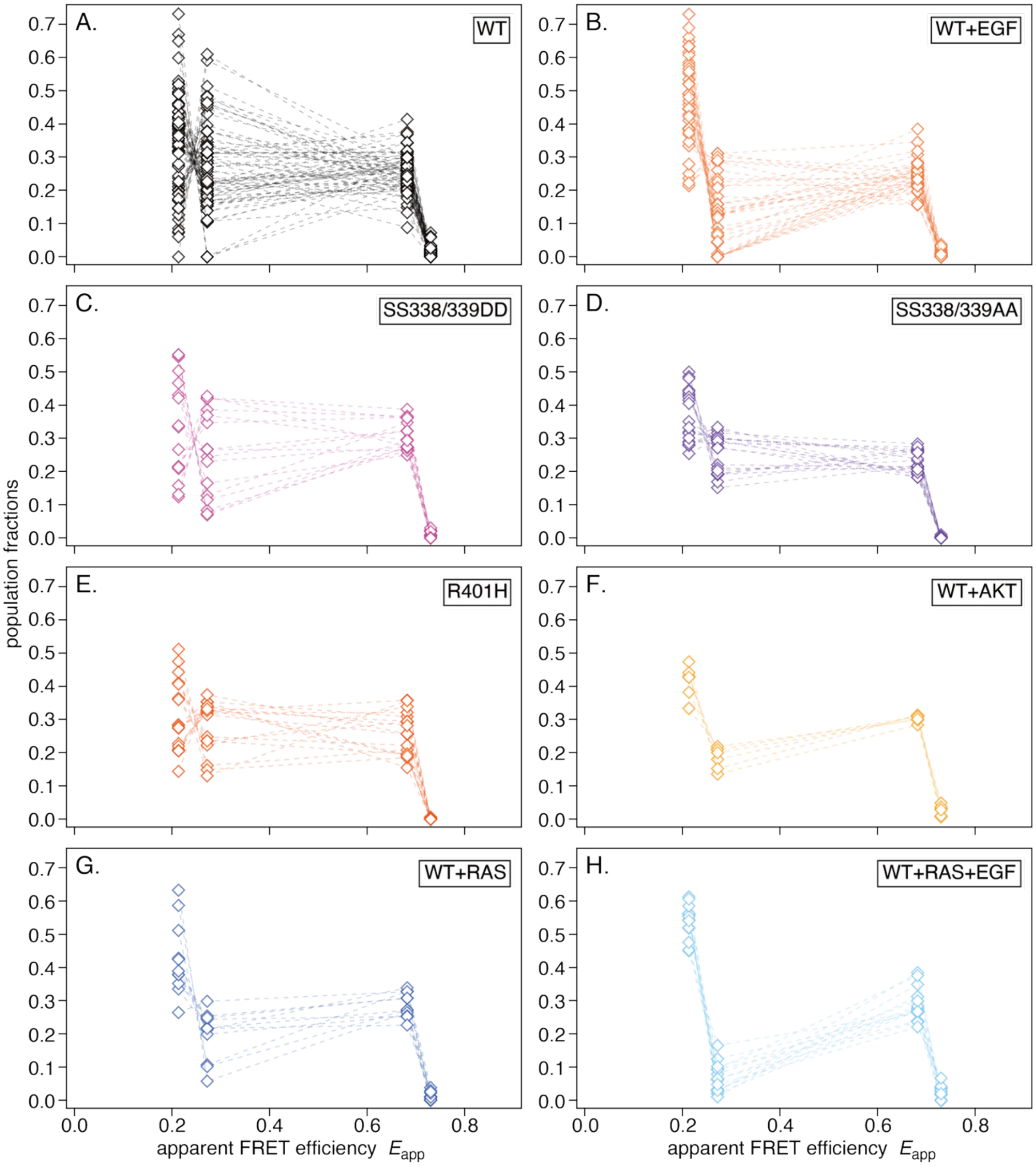

Supplementary Figure S3. Component populations obtained by fitting to individual cells for **(A)** WT, **(B)** WT+EGF, **(C)** SS338/339DD, **(D)** SS338/339AA, **(E)** R401H, **(F)** WT+AKT, **(G)** WT+RAS, and **(H)** WT+RAS+EGF. Dashed lines connect points for the same cell. **(A)** is identical to Fig.4A of the main text.

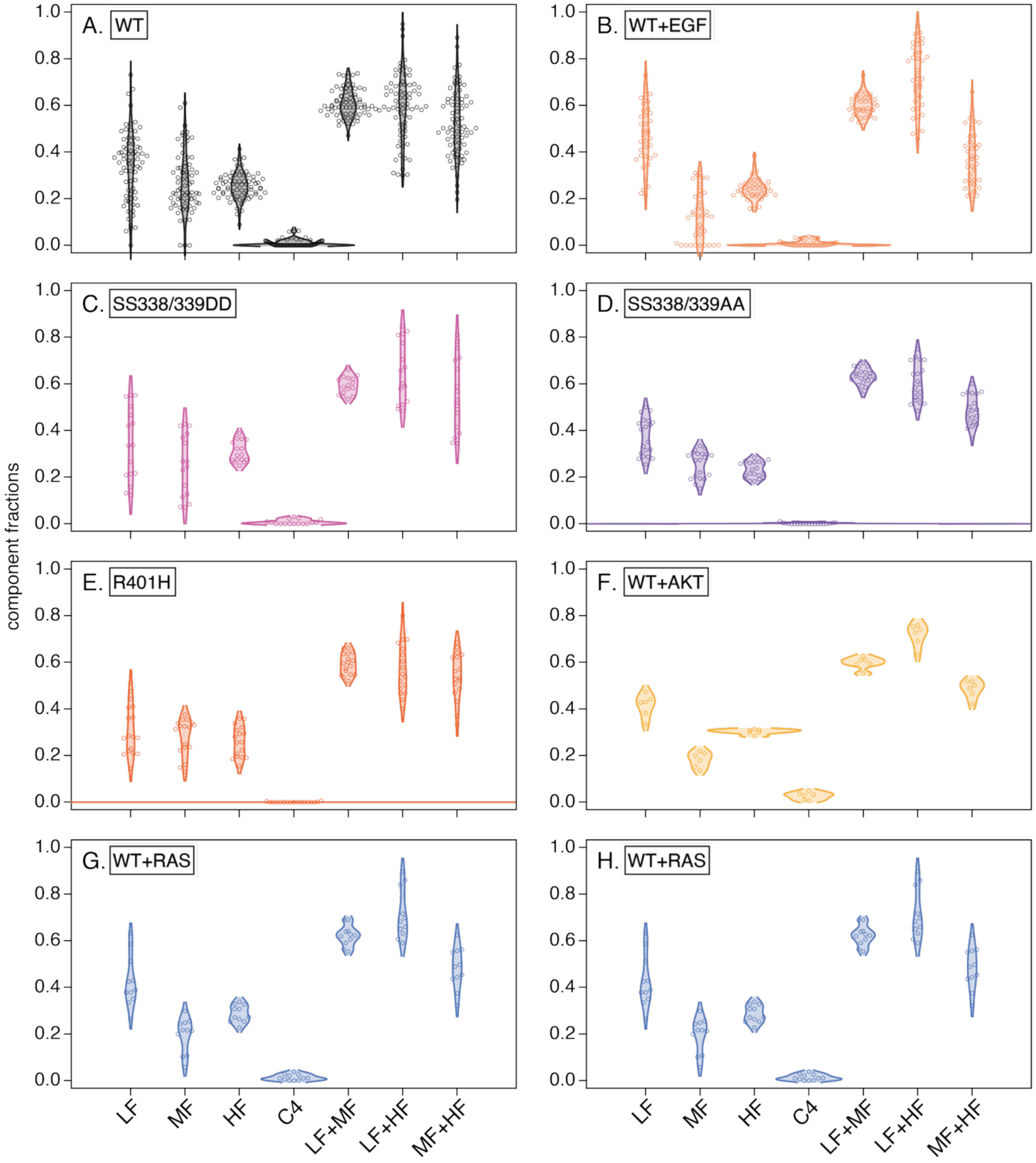

Supplementary Figure S4. Distributions of component populations obtained by fitting to individual cells for **(A)** WT, **(B)** WT + EGF, **(C)** SS338/339DD, **(D)** SS338/339AA, **(E)** R401H, **(F)** WT+AKT, **(G)** WT+RAS, and **(H)** WT+RAS+EGF. Four components (LF, MF, HF, and C4) and selected sums (LF+MF, LF+HF, MF+HF). LF+MF and HF commonly have small variances. **(A)** is identical to Fig.4B of the main text.

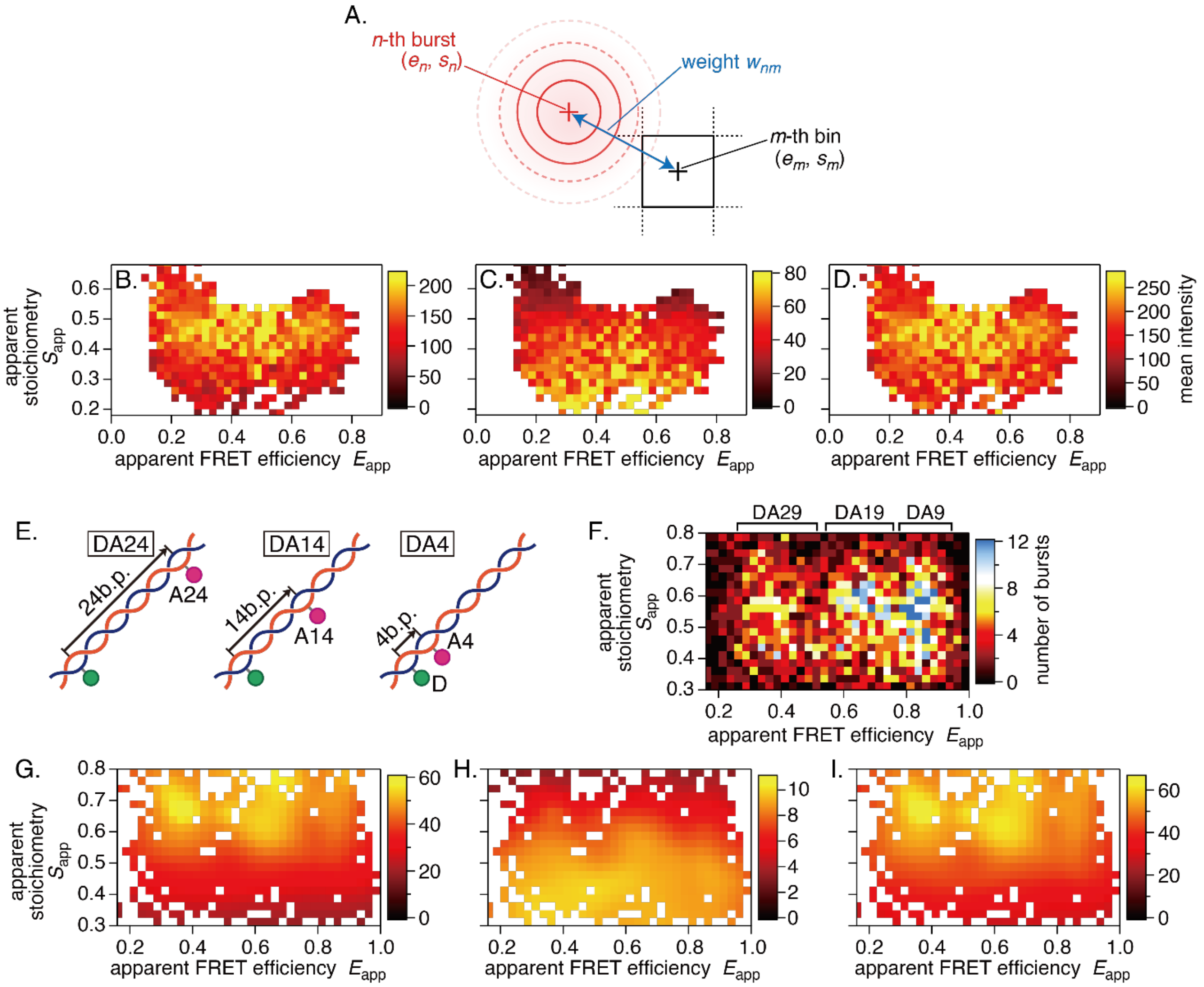

Supplementary Figure S5. Burst intensity analysis. **(A)** Principle of mean intensity calculation. To calculate the mean intensity of a certain bin, all bursts, including those out of the bin, are averaged with the weight. Mean burst intensity of **(B)** donor-excited, **(C)** acceptor-excited fluorescence, and **(D)** their sum, calculated as the simple mean of bursts within each bin. **(E)** Three double-stranded DNAs as reference samples for monomers with different FRET. **(F)** Burst distribution of those mixtures on the *E-S* map, and the weighted mean burst intensity of **(G)** donor-excited, **(H)** acceptor-excited fluorescence, and **(I)** their sum.

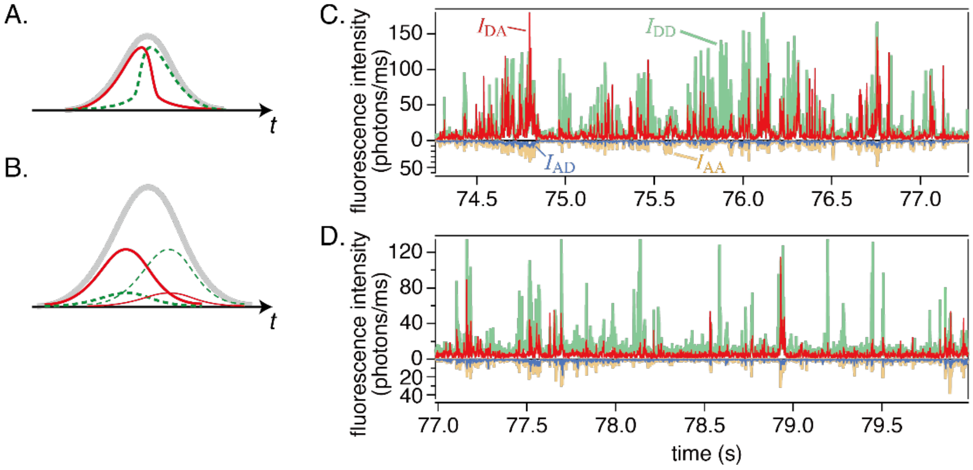

Supplementary Figure S6. Illustrations of **(A)** intraburst state transition and **(B)** successively detected double bursts. Red solid and green dashed lines represent acceptor and donor fluorescence, respectively. The thick gray line represents the total intensity. While single-molecule and double-molecule bursts are difficult to distinguish from the fluorescence change, differences appear in the burst intensity in mean. Fluorescence time traces of the measurement of **(C)** Fig.5E and **(D)** Fig.5F of the main text are examples of a high and low concentration sample, respectively.

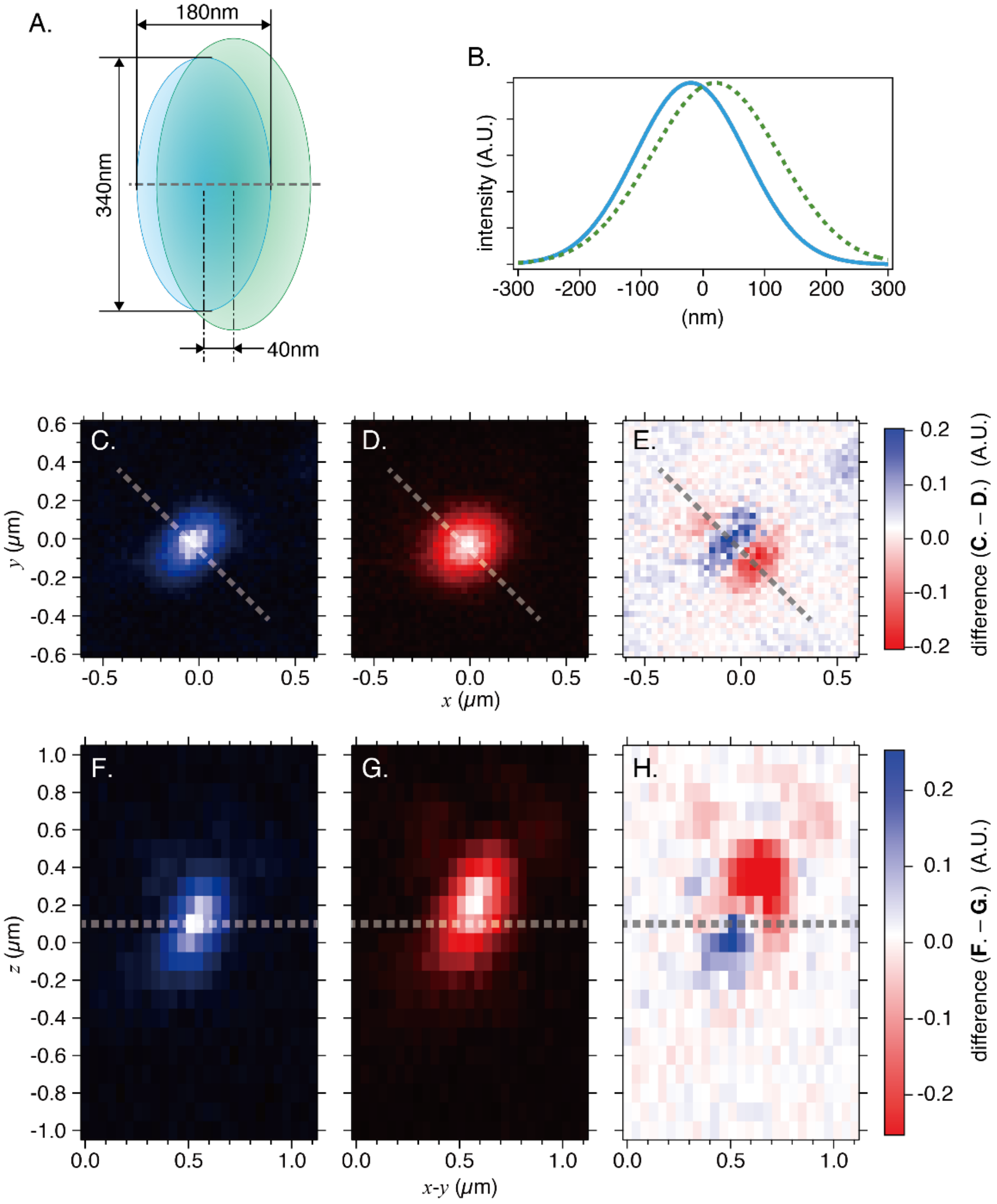

Supplementary Figure S7. (A) Two PSFs of different wavelengths (488/561 nm) with a spatial displacement. The ellipses represent the σ of the 3D Gaussian function; the PSF for 561 nm is larger by the wavelength ratio. Dimensions are used for simulation. **(B)** Horizontal cross-section at the position of the dashed line in **(A)**. **(C)–(H)** Three-dimensional fluorescence imaging of a fluorescent bead. **(C)–(E)** Horizontal and **(F)–(H)** vertical cross-section at the position of the dashed lines in **(C)**-**(E)** and **(F)–(H)**, respectively. **(C)**, **(F)** and **(D)**, **(G)** are fluorescence images excited by 488 nm and 561 nm light, respectively, and **(E)** and **(H)** are the difference between them. Spatial displacement and size difference can be seen. The tilt of PSF in **(F)–(H)** is attributed to thermal drift during *z*-scanning.

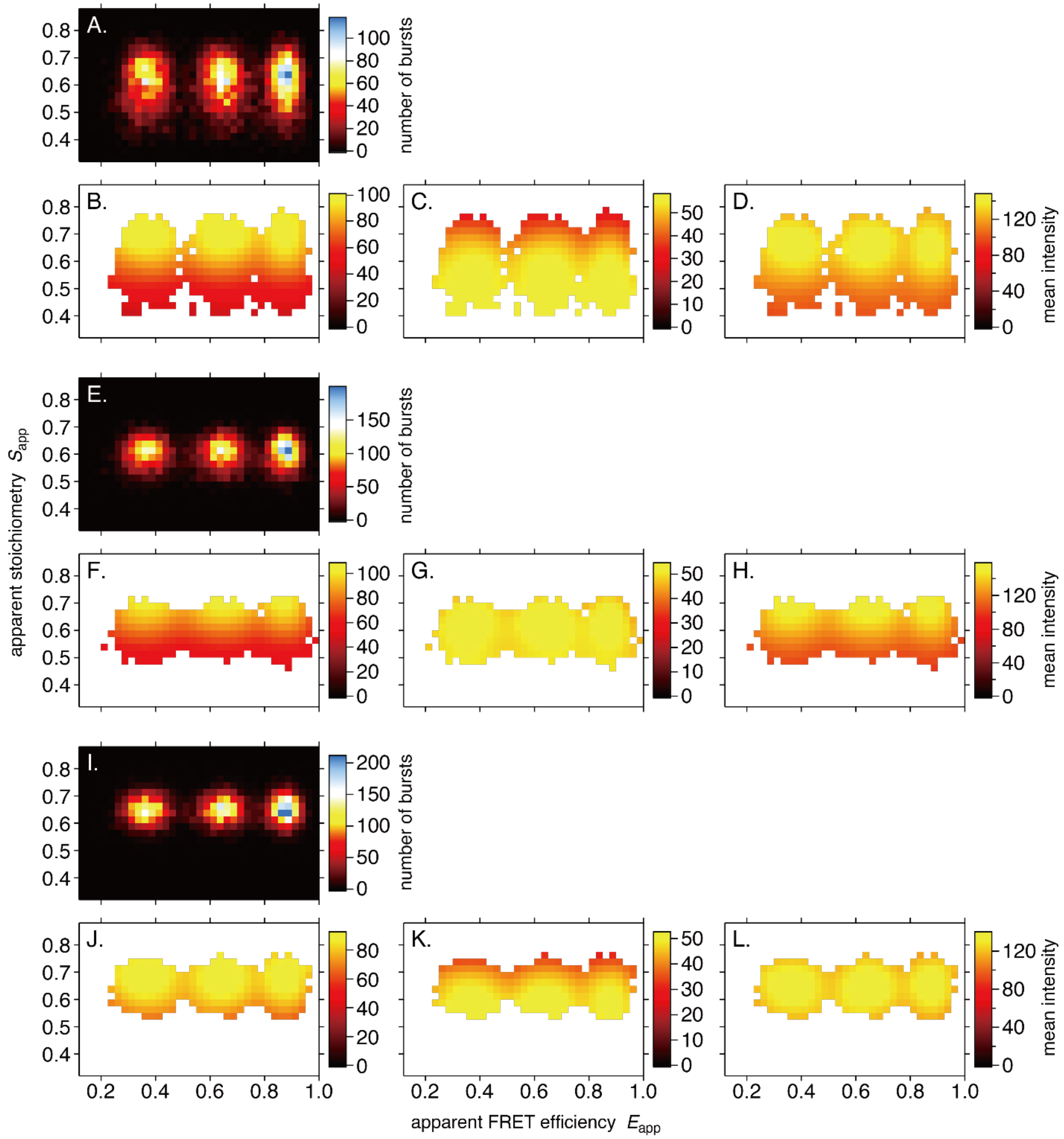

Supplementary Figure S8. Simulation results of the weighted mean burst intensity distributions with PSF displacement. Burst distribution, donor-excited fluorescence intensity, acceptor-excited fluorescence intensity, and their sum, respectively, for conditions **(A)–(D)** PSF displacement + PSF size difference, **(D)–(H)** PSF size difference with no PSF displacement, and **(I)–(L)** perfectly overlapped PSFs (no displacement and size difference between PSFs).
